# Middle Latency Response Study of Auditory Evoked Potentials' Amplitudes and Lantencies Audiologically Normal Individuals

**DOI:** 10.1016/S1808-8694(15)31125-3

**Published:** 2015-10-20

**Authors:** Ivone Ferreira Neves, Isabela Crivellaro Gonçalves, Renata Aparecida Leite, Fernanda Cristina Leite Magliaro, Carla Gentile Matas

**Affiliations:** 1 PhD in Sciences - FMUSP, Speech and Hearing Therapist of the Speech and Hearing Therapy Course - FMUSP; 2 Postgraduate student in Clinical Audiology - FMABC, Scholarship holder - FAPESP; 3 M.S in Sciences - FMUSP, Speech Therapist; 4 M.S in Sciences - FMUSP, Speech Therapist; 5 PhD in Human Communication Disorders - EPM/UNIFESP, Professor at the Speech and Hearing Therapy Course - FMUSP

**Keywords:** auditory perception, auditory evoked potentials, hearing tests

## Abstract

**Summary:**

Contemporary cohort cross-sectional study. Introduction: The auditory middle latency response (AMLR) is generated between 10 and 80 ms and has multiple generators, with a greater contribution from the thalamus-cortical pathways. The establishment of normality criteria for latency and amplitude values is necessary for clinical use.

**Aim:**

to analyze the latency and amplitude of the AMLR in individuals without audiological disorders, and verify the reliability of Pa-Nb amplitude.

**Materials and Methods:**

The AMLR of 25 individuals was collected during 2005 and the Na, Pa, Nb components were analyzed for each tested ear (A1 and A2), and electrode positioning (C3 and C4).

**Results:**

A statistically significant difference was noticed among middle latency values for C3A1 and C4A1 regarding components Na and Pa, and no difference for component Nb and mean values for amplitudes Na-Pa and Pa-Nb. Conclusions: We established average and standard deviation values for latency and amplitude parameters for components Na, Pa, Nb and Na-Pa and Pa-Nb, under conditions C3A1, C4A1, C3A2, C4A2, providing a parameter for the analysis and interpretation of this potential.

## INTRODUCTION

The use of Auditory Evoked Potentials (AEP) to study the Central Auditory Nervous System has been increasingly used and of great help in audiologic differential diagnosis.

As they described the clinical advantages of AEP, some authors mention especially the fact that these are accurate and objective tests, independent from an individual's subjective response, and it may be very useful in the evaluation of children with language disorders and also in monitoring therapeutic process because of the very plasticity of the nervous system. Moreover, they stress the fact that it bears high correlation to physiological changes in the auditory pathway and is efficient in distinguishing lesions from functional alterations in the Central Nervous System (CNS)^1^.

One of the most used classifications for the AEP is related to the latency period in which they appear. Thus, they may be classified as early, middle or late^2^.

The Middle Latency Auditory Evoked Potential (MLAEP) is made up of a set of positive (“P” waves) and negative (“N” waves) waves; already described by Goldstein and Rodman in 1967^3^. The first MLAEP negative wave was called Na, followed by the positive wave Pa and, by the Nb, Pb and, sometimes, Nc and Pc waves, being the Pa the most constant and most frequently used. This potential occurs between 10 and 80 milliseconds (ms) after the acoustic stimulus onset^4^, and seems to have multiple generators, with a greater contribution of the thalamus-cortical pathways, and less contribution from the inferior colliculus and the reticular formation^5^.

MLAEP is a sensitive potential for low frequencies, and the difference found between the behavioral auditory threshold and the electrophysiological threshold is of approximately 10 dB^6^.

Studies have shown higher amplitude and latency values for children when compared to adults; and more similar values tend to appear at ages between 8 and 10 years7. Besides maturity-related factors, MLAEP responses are also influenced by gender - latency values are higher for males and amplitude values are higher for females. They are also influenced by sleep and sedation7.

The morphology of such potentials is clinically important, and one should confirm the presence of a long negative peak (Na), followed by a positive peak (Pa), between 15 and 30ms, besides wave reproducibility. Although the Pa wave is the dominant component, its morphology may vary substantially from individual to individual and also between the ears and electrodes in the same individual^7^.

Within normal values there may be different types of possible morphologies for the Na, Pa, Nb and Pb values: single peaks for Pa and Pb, different by a mild slope between them; broad Pa with a double peak and short slope between them, and the second peak has lower latency than what is expected for Pb; Pa and Pb, separated by Nb; evident Pa with no Pb^8^.

As in any other AEP, response analysis criteria are in function of the latency (milliseconds - ms) and amplitude (microvolt - μv) values, and an intensity reduction causes an increase in latency values and reduction in amplitude values. Studies suggest that CNS alterations would affect more the amplitude than the latency values. Amplitude also seems to be the best indicator of functional alterations, since latency values bear large variations even among normal individuals. In general, the measured amplitude is generated between peaks Na and Pa, since it is the most robust. Using the Pa-Nb amplitude value may be one option if it is not possible to attain the Na-Pa amplitude value^7^.

MLAEP is one of the most promising for the identification of alterations in the central nervous system. Nonetheless, its little clinical use is due to the fact that there may be a great variability in latency and amplitude values among subjects^9^, and this makes it difficult to establish values of normality, thus making it necessary to research this.

Matas et al. (1994)^10^ were among the first Brazilian authors to study latency values for MLAEP waves in normal individuals. The authors found statistically significant differences among latency values for each ear, and justified the diversity of values encountered in the literature because of the different methods employed.

Thus, studies aiming at standardizing the parameters employed in carrying out the test and the response analysis for normal individuals are still relevant in order to have a better clinical use for the MLAEP.

## OBJETIVE

The goal of the present investigation was to analyze latency values for waves Na, Pa and Nb and amplitude values for waves Na-Pa and Pa-Nb from the MLAEP in adult individuals without hearing alterations, as well as to check the Pa-Nb amplitude value reliability by comparing Na-Pa and Pa-Nb amplitude values.

## MATERIALS AND METHODS

This study was approved by the Ethics Committee of the Institution where it was carried out under protocol #008/2006. Data obtained in the evaluations were used only after each participant signed an informed consent form. We analyzed the MLAEP traces from 25 individuals, 20 females and 5 males, with ages ranging between 19 and 24 years.

All the patients were voluntary and had university degrees, without prior history of neurologic or psychiatric disorders and presented normal results in the tests that make up basic audiologic investigation (Tonal audiometry with hearing thresholds up to 20 dB HL at the 250, 500, 1k, 2k, 3k, 4k, 6k and 8k Hz frequencies^11^; Speech recognition threshold (SRT) equal to the average of frequencies 500, 1k and 2k Hz or up to 10 dB and above; Percentage Index of Speech Recognition (PISR) equal or above 88%^12^; Acoustic immitance measures with normal tympanometric curves - type A - and ipsilateral and contralateral acoustic reflexes present at normal levels in the frequencies of 500, 1k, 2k and 4k Hz^13^).

In order to carry out basic auditory evaluation, we used the following equipment: middle ear analyzer from Grason-Stadler, model GSI-33 (ANSI S3.39 - 1987)^14^; Sound-proof booth with environmental noise within the ANSI S3.1 - 1991^15^ standard; Grason-Stadler model GSI-61 audiometer and TDH-50 supra-aural headphones (ANSI S3.6 - 1989)^16^. For MLAEP acquisition we used a Traveller-Express Bio-logic electroneuromyograph, with the EP317 (ANSI S3.7 - 1996)^17^program.

The procedures used were: anamnesis, otoscopy, tympanometry, ipsi and contralateral acoustic reflexes in the frequencies of 500, 1k, 2k and 4k Hz, Tonal Audiometry in the frequencies of 250, 500, 1k, 2k, 3k, 4k, 6k and 8k Hz, SRT, SRPI and MLAEP.

The acoustic stimulus parameters used for MLAEP acquisition were: click-type stimulus, in a single ear, at 70 dB HL, at 9.9/s presentation speed, making up a total of 1,000 clicks. The electrodes were placed in A1 (left mastoid), A2 (right mastoid), C3 (left temporo-parietal junction), C4 (right temporo-parietal junction) e Cz (vertex). The responses recorded were from the evoked potentias found in C3-A1, C4-A1, C3-A2 and C4-A2; latency values for components Na, Pa and Nb and amplitude values for Na-Pa and Pa-Nb were analyzed.

MLAEP data were submitted to statistical analysis using the ANOVA average comparison test and the Confidence Interval method in order to complement the descriptive analysis. In order to analyze the comparisons carried out, we used the p-value, adopting a significance level of 0.05 (5%), with a 95% confidence interval.

The statistical analysis was carried out in relation to the latency and amplitude average values for components Na, Pa and Nb for four modalities: C3-A1, C4-A1, C3-A2 and C4-A2.

## RESULTS

The values presented on Tables 1, 2, 3, 4, 5 and 6 correspond to the averages, medians, standard deviations, minimum and maximum limits and p-values for the Na, Pa and Nb components latency values and amplitudes for Na-Pa and Pa-Nb.

**Table 1:** Means, medians, standard deviations, maximum and minimum latency limits of the Na component in positions C3-A1, C4-A1, C3-A2 and C4-A2, of the individuals studied.

Latency (ms)	Na			
	C3-A1	C4-A1	C3-A2	C4-A2
Mean	20,77	23,17	21,62	21,12
Median	19,89	23,40	20,28	19,50
Standard Deviation	3,12	4,84	3,96	4,58
Minimum	15,60	15,21	16,77	12,48
Maximum	27,30	30,81	30,03	32,76
p-value	0,037*		0,682

**Table 2:** Means, medians, standard deviations, maximum and minimum latency limits of the Pa component in positions C3-A1, C4-A1, C3-A2 and C4-A2, of the individuals studied.

Latency (ms)	Pa			
	C3-A1	C4-A1	C3-A2	C4-A2
Mean	31,07	32,85	31,00	31,75
Median	31,98	33,15	31,98	31,59
Standard Deviation	2,86	3,84	4,14	4,05
Minimum	23,79	24,96	23,40	22,62
Maximum	33,93	39,78	37,83	40,56
p-value	0,063#		0,517

**Table 3:** Means, medians, standard deviations, maximum and minimum latency limits of the Nb component in positions C3-A1, C4-A1, C3-A2 and C4-A2, of the individuals studied.

Latency (ms)	Nb			
	C3-A1	C4-A1	C3-A2	C4-A2
Mean	41,99	42,67	41,62	41,70
Median	42,12	42,90	43,29	41,73
Standard Deviation	5,92	5,26	5,62	4,35
Minimum	24,12	28,86	28,47	28,08
Maximum	50,70	51,87	49,53	49,53
p-value	0,668		0,955

**Table 4:** Means, medians, standard deviations, maximum and minimum latency limits of the Na-Pa amplitude in positions C3-A1, C4-A1, C3-A2 and C4-A2, of the individuals studied.

Amplitudes (*μ*v)	NaPa			
	C3-A1	C4-A1	C3-A2	C4-A2
Mean	1,38	1,40	1,42	1,78
Median	1,10	1,06	0,92	1,11
Standard Deviation	0,99	1,17	1,61	2,22
Minimum	0,24	0,29	0,20	0,28
Maximum	3,57	5,11	8,58	9,90
p-value	0,948		0,512

**Table 5:** Means, medians, standard deviations, maximum and minimum latency limits of the Pa-Nb amplitude in positions C3-A1, C4-A1, C3-A2 and C4-A2, of the individuals studied.

Amplitudes (*μ*v)	Pa-Nb			
	C3-A1	C4-A1	C3-A2	C4-A2
Mean	1,27	1,21	1,36	1,33
Median	1,13	1,19	1,11	0,96
Standard Deviation	0,66	0,66	0,97	1,29
Minimum	0,42	0,11	0,11	0,07
Maximum	2,67	2,42	5,21	6,49
p-value	0,748		0,926

**Table 6:** Comparison among the Na-Pa and Pa-Nb amplitude averages in positions C3-A1, C4-A1, C3-A2 and C4-A2, of the individuals studied.

Amplitudes (*μ*v)	Pa-Na X Pa-Nb			
	C3-A1	C4-A1	C3-A2	C4-A2
Na-Pa Mean	1,38	1,40	1,42	1,78
Pa-Nb Mean	1,27	1,21	1,36	1,33
p-value	0,644	0,479	0,873	0,381

The data depicted on Table 1 indicate a statistically significant difference among modalities C3-A1 and C4-A1, for the Na component latency value.

On Table 2 we can see that although there has been no statistically significant difference among the different modalities for the Pa component latency value, the difference among modalities C3-A1 and C4-A1 may be deemed biased, since the p-value was very close to the acceptable limit (0.05).

According to Table 3, there was no statistically significant difference among the modalities studied for the Nb Component latency.

Regarding Na-Pa (Table 4) and Pa-Nb (Table 5) amplitude values, we noticed that there was no statistically significant difference among the modalities studies.

By comparing mean values of the Na-Pa and Pa-Nb amplitudes, we may see that there was no statistically significant difference among them (Table 6).

## DISCUSSION

### Na, Pa and Nb MLAEP latency values

Latency values for components Na, Pa and Nb, found in the present study were compared to those from other studies, as shown in Chart 1. In the present study we analyzed the latencies in the C3 and C4 electrode positions for each ear, and this was not done in the other studies (Tables 1, 2 and 3).

Latency values found for Na and Pa were more similar to those found by Purdy et al. (2002)^18^ and by Frizzo (2004)^19^, although we did not have greater differences when compared to other studies, thus favoring a normal reference value (Tables 1 and 2).

The largest differences in values found among the authors were seen for the Nb component. In this potential, the Nb component is the most distal one and, very likely because it is an upper area in the auditory pathway, it may be difficult to see, thus making it harder to establish a more accurate normality value (Table 3).

In the present investigation, as we can see on Table 1, we found a statistically significant difference among latency values in positions C3 and C4 for the Na component, and this was not seen in the other studies. This suggests that the mean values found must be differentiated according to electrode positioning, that is, for C3-A1, C4-A1 conditions.

According to Table 1, for the three components, the median values found were very close to the mean values, thus suggesting that the sample was symmetrical in regards of the Na, Pa and Nb component latencies. This means that, approximately 50% of the individuals presented values below the average, and 50% were above it.

The values found for the standard deviation were small, thus indicating a low result variability in relation to mean values, in other words, most individuals presented latency values within the average with standard deviation.

Moreover, in the results found for Na, Pa and Nb, we noticed a large gap among minimum and maximum values, suggesting that, even in individuals without alterations or auditory complaints there may be discrepant latency values. Thus, studies with normal individuals, in a larger sample, would be needed in order to indicate a more accurate range of normal values. According to McGee et al. (1988)^8^, there may be numerous variations within MLAEP normal values, and this variation is one of the factors that make it difficult to establish normality criteria. Schochat (2001)^20^ also reported this value variability for such potential in his study.

Even then, with the data from the present study, it is possible to conclude that the mean values, with the standard deviations found for Na, Pa and Nb latencies, for both electrode positions (C3 and C4) and both ears (A1 and A2), may serve as normality criteria in order to analyze the MLAEP curve, considering the possibility that, in some cases, discrepant values may be found.

### Na-Pa and Pa-Nb Amplitude Values

Values for the MLAEP amplitude were recorded for two measures: Na-Pa and Pa-Nb, as depicted on Tables 4 and 5.

The comparison of MLAEP amplitude values with other studies are depicted on Chart 2.

The values found for the Na-Pa amplitude were similar to those found by other studies, and even closer to those found by Purdy et al. (2002)^18^.

As we see on Tables 4 and 5, we did not find statistically significant differences in electrode positions C3 and C4. May be one single mean value could be established for amplitude in each ear.

Differently from what was seen for latency values, the median was not very close to the mean value, thus indicating an asymmetrical sampling. Concurrently, standard deviations were very close to the mean values, indicating a high result variability. Moreover, we noticed extremely discrepant minimum and maximum values (varying between 0.2 and 9.9μv and between 0.07 and 6.49μv). This means that a broad range of values may be considered within the normal range and, consequently, makes it difficult to define response patterns for the MLAEP curve analysis as far as amplitude is concerned. More numerous studies would also be necessary in order to better define normality criteria for the MLAEP amplitude values.

Thus, the amplitude measure could be further indicated for the intra-subject comparison than the inter-subject comparison, as it happens in therapeutic monitoring situations.

Most studies analyzed amplitude according to the Na-Pa component, and it was only the present study and the one carried out by Frizzo (2004)^19^ that also analyzed the Pa-Nb amplitude. According to Hall III (1992)^7^, and later confirmed by Frizzo (2004)19, the Na-Pa component would be the most robust one and the one more visible.

In the present investigation, the Na-Pa amplitude was also the one better identified and that presented the highest values (1.39μv for A1 and 1.59μv for A2), as we can see on Table 6, although there was no statistically significant difference between such measure and the Pa-Nb (1.24μv for A1 and 1.34μv for A2). These results suggest that the Na-Pa amplitude would be the one most suited for MLAEP, also considering the fact that the Nb component latency bears great variability. Hall III (1992)^7^ stated that the amplitude to be measured at the MLAEP should be the one generated between the Na and Pa peaks, since it is the most robust one, and the Pa-Nb amplitude value may be used if there is no Na-Pa.

In the present study, the Na, Pa, and Nb component latency measures were better in order to define normality criteria when compared to amplitudes, due to the variability found in the results.

Such data was not in agreement to the one found by Hall III (1992)^7^, who stated that both CNS disorders and functional alterations impact more the amplitude than the latency values. Different authors (Purdy et al., 2002^18^; Schochat et al., 2004^9^) also stated that they used Na-Pa amplitude values more than latency ones as normality criteria.

## CONCLUSION

In this study, we analyzed the latency values for components Na, Pa and Nb and the Na-Pa and Pa-Nb amplitudes of the MLAEP electrophysiological potential in 25 individuals without audiologic abnormalities, in order to establish normality criteria.

For latency we obtained the following mean values:

For Na (ms): 20.77 (C3-A1), 23.17 (C4-A1), 21.62 (C3-A2) and 21.12 (C4-A2);

For Pa (ms): 31.07 (C3-A1), 32.85 (C4-A1), 31.00 (C3-A2) and 31.75 (C4-A2);

For Nb (ms): 41.99 (C3-A1), 42.67 (C4-A1), 41.62 (C3-A2) and 41.70 (C4-A2).

For amplitude we obtained the following mean values:

For Na-Pa (μv): 1.38 (C3-A1), 1.40 (C4-A1), 1.42 (C3-A2) and 1.78 (C4-A2);

For Pa-Nb (μv): 1.27 (C3-A1), 1.21 (C4-A1), 1.36 (C3-A2) and 1.33 (C4-A2).

We did not see any significant difference between the mean values for Na-Pa and Pa-Nb amplitudes, suggesting that the Pa-Nb amplitude may be considered a reliable parameter for MLAEP analysis.

Establishing normality criteria for MLAEP is of paramount importance, having seen that such potential is being increasingly more used clinically in order to determine more accurate audiologic diagnosis.
